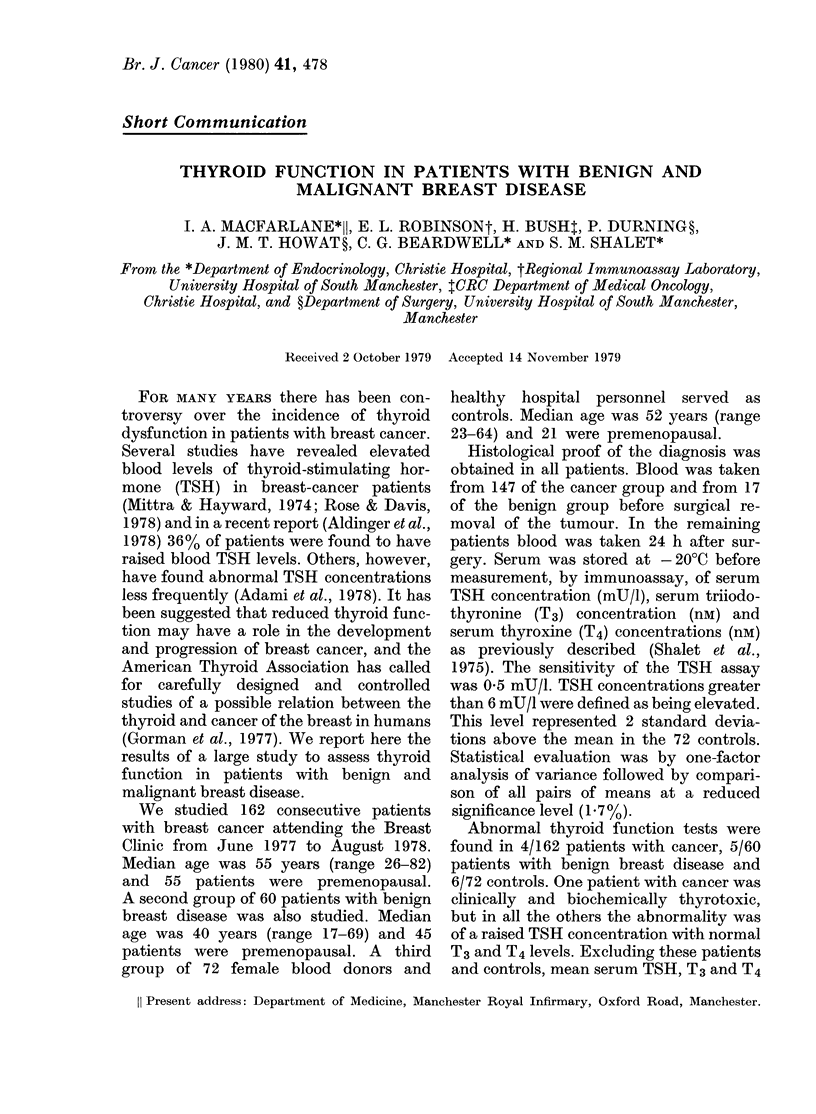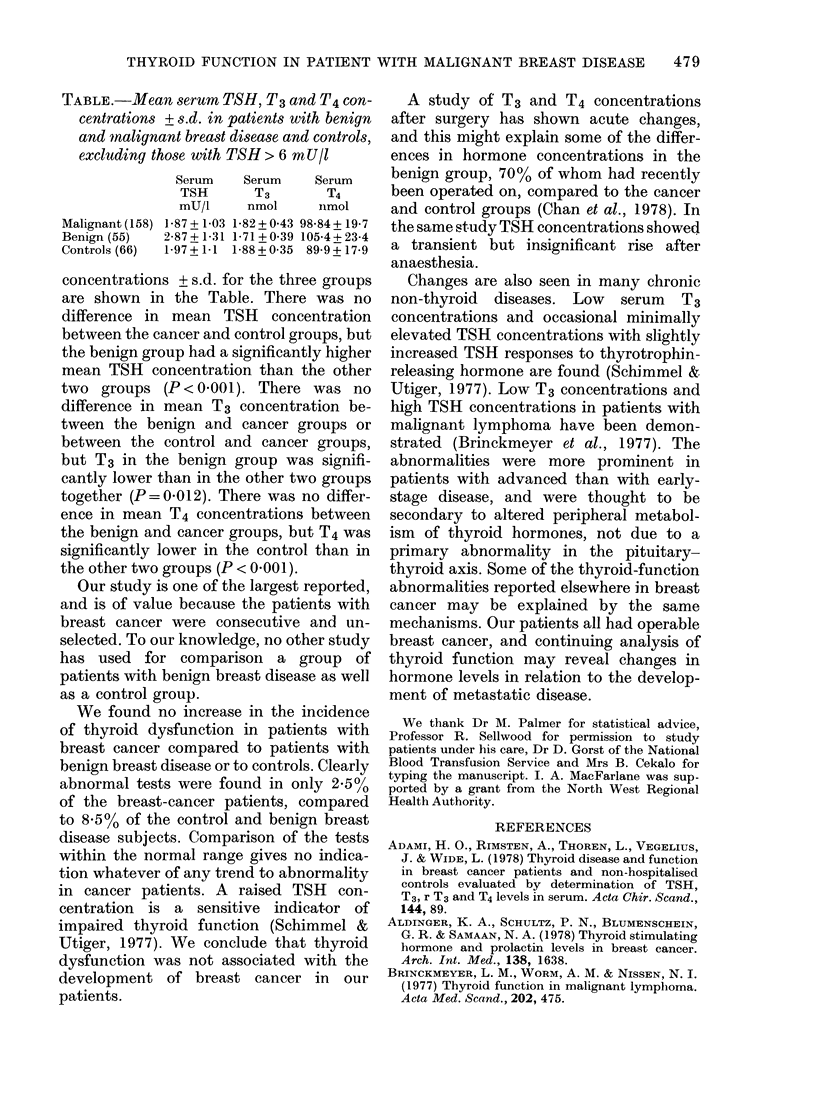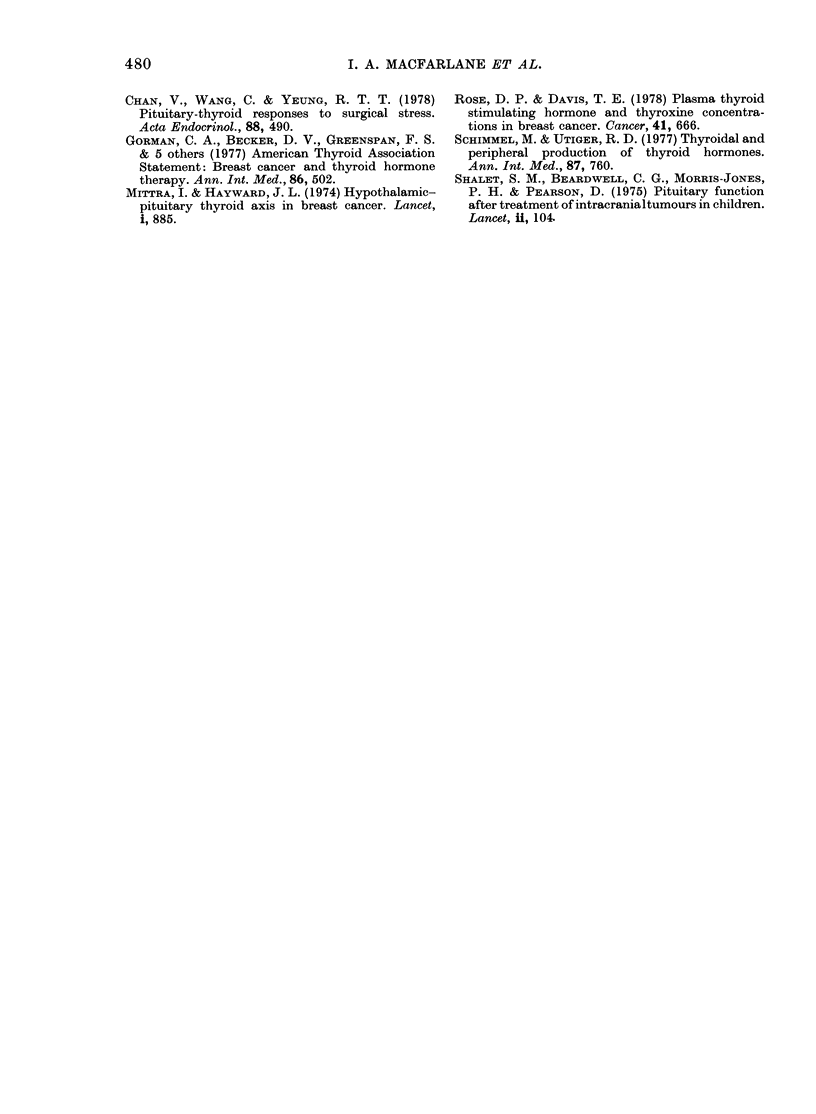# Thyroid function in patients with benign and malignant breast disease.

**DOI:** 10.1038/bjc.1980.73

**Published:** 1980-03

**Authors:** I. A. MacFarlane, E. L. Robinson, H. Bush, P. Durning, J. M. Howat, C. G. Beardwell, S. M. Shalet


Br. J. Cancer (1980) 41, 478

***Short Communication***

### THYROID FUNCTION IN PATIENTS WITH BENIGN AND MALIGNANT BREAST DISEASE

I. A. MACFARLANE*||, E. L. ROBINSON†, H. BUSH‡, P. DURNING§, J. M. T. HOWAT§, C. G. BEARDWELL* AND S. M. SHALET*

*From the *Department of Endocrinology, Christie Hospital, †Regional Immunoassay Laboratory, University Hospital of South Manchester, ‡CRC Department of Medical Oncology, Christie Hospital, and §Department of Surgery, University Hospital of South Manchester, Manchester*

Received 2 October 1979 Accepted 14 November 1979

FOR MANY YEARS there has been controversy over the incidence of thyroid dysfunction in patients with breast cancer. Several studies have revealed elevated blood levels of thyroid-stimulating hormone (TSH) in breast-cancer patients (Mittra & Hayward, 1974; Rose & Davis, 1978) and in a recent report (Aldinger *et al.*, 1978) 36% of patients were found to have raised blood TSH levels. Others, however, have found abnormal TSH concentrations less frequently (Adami *et al.*, 1978). It has been suggested that reduced thyroid function may have a role in the development and progression of breast cancer, and the American Thyroid Association has called for carefully designed and controlled studies of a possible relation between the thyroid and cancer of the breast in humans (Gorman *et al.*, 1977). We report here the results of a large study to assess thyroid function in patients with benign and malignant breast disease.

We studied 162 consecutive patients with breast cancer attending the Breast Clinic from June 1977 to August 1978. Median age was 55 years (range 26–82) and 55 patients were premenopausal. A second group of 60 patients with benign breast disease was also studied. Median age was 40 years (range 17–69) and 45 patients were premenopausal. A third group of 72 female blood donors and healthy hospital personnel served as controls. Median age was 52 years (range 23–64) and 21 were premenopausal.

Histological proof of the diagnosis was obtained in all patients. Blood was taken from 147 of the cancer group and from 17 of the benign group before surgical removal of the tumour. In the remaining patients blood was taken 24 h after surgery. Serum was stored at −20°C before measurement, by immunoassay, of serum TSH concentration (mU/l), serum triiodothyronine ($T_3$) concentration (nM) and serum thyroxine ($T_4$) concentrations (nM) as previously described (Shalet *et al.*, 1975). The sensitivity of the TSH assay was 0·5 mU/l. TSH concentrations greater than 6 mU/l were defined as being elevated. This level represented 2 standard deviations above the mean in the 72 controls. Statistical evaluation was by one-factor analysis of variance followed by comparison of all pairs of means at a reduced significance level (1·7%).

Abnormal thyroid function tests were found in 4/162 patients with cancer, 5/60 patients with benign breast disease and 6/72 controls. One patient with cancer was clinically and biochemically thyrotoxic, but in all the others the abnormality was of a raised TSH concentration with normal $T_3$ and $T_4$ levels. Excluding these patients and controls, mean serum TSH, $T_3$ and $T_4$

|| Present address: Department of Medicine, Manchester Royal Infirmary, Oxford Road, Manchester.

TABLE.—*Mean serum TSH, $T_3$ and $T_4$ concentrations ± s.d. in patients with benign and malignant breast disease and controls, excluding those with TSH > 6 mU/l*

| | Serum TSH mU/l | Serum $T_3$ nmol | Serum $T_4$ nmol |
|---|---|---|---|
| Malignant (158) | 1·87 ± 1·03 | 1·82 ± 0·43 | 98·84 ± 19·7 |
| Benign (55) | 2·87 ± 1·31 | 1·71 ± 0·39 | 105·4 ± 23·4 |
| Controls (66) | 1·97 ± 1·1 | 1·88 ± 0·35 | 89·9 ± 17·9 |

concentrations ± s.d. for the three groups are shown in the Table. There was no difference in mean TSH concentration between the cancer and control groups, but the benign group had a significantly higher mean TSH concentration than the other two groups ($P < 0·001$). There was no difference in mean $T_3$ concentration between the benign and cancer groups or between the control and cancer groups, but $T_3$ in the benign group was significantly lower than in the other two groups together ($P = 0·012$). There was no difference in mean $T_4$ concentrations between the benign and cancer groups, but $T_4$ was significantly lower in the control than in the other two groups ($P < 0·001$).

Our study is one of the largest reported, and is of value because the patients with breast cancer were consecutive and unselected. To our knowledge, no other study has used for comparison a group of patients with benign breast disease as well as a control group.

We found no increase in the incidence of thyroid dysfunction in patients with breast cancer compared to patients with benign breast disease or to controls. Clearly abnormal tests were found in only 2·5% of the breast-cancer patients, compared to 8·5% of the control and benign breast disease subjects. Comparison of the tests within the normal range gives no indication whatever of any trend to abnormality in cancer patients. A raised TSH concentration is a sensitive indicator of impaired thyroid function (Schimmel & Utiger, 1977). We conclude that thyroid dysfunction was not associated with the development of breast cancer in our patients.

A study of $T_3$ and $T_4$ concentrations after surgery has shown acute changes, and this might explain some of the differences in hormone concentrations in the benign group, 70% of whom had recently been operated on, compared to the cancer and control groups (Chan *et al.*, 1978). In the same study TSH concentrations showed a transient but insignificant rise after anaesthesia.

Changes are also seen in many chronic non-thyroid diseases. Low serum $T_3$ concentrations and occasional minimally elevated TSH concentrations with slightly increased TSH responses to thyrotrophin-releasing hormone are found (Schimmel & Utiger, 1977). Low $T_3$ concentrations and high TSH concentrations in patients with malignant lymphoma have been demonstrated (Brinckmeyer *et al.*, 1977). The abnormalities were more prominent in patients with advanced than with early-stage disease, and were thought to be secondary to altered peripheral metabolism of thyroid hormones, not due to a primary abnormality in the pituitary–thyroid axis. Some of the thyroid-function abnormalities reported elsewhere in breast cancer may be explained by the same mechanisms. Our patients all had operable breast cancer, and continuing analysis of thyroid function may reveal changes in hormone levels in relation to the development of metastatic disease.

We thank Dr M. Palmer for statistical advice, Professor R. Sellwood for permission to study patients under his care, Dr D. Gorst of the National Blood Transfusion Service and Mrs B. Cekalo for typing the manuscript. I. A. MacFarlane was supported by a grant from the North West Regional Health Authority.

### REFERENCES

ADAMI, H. O., RIMSTEN, A., THOREN, L., VEGELIUS, J. & WIDE, L. (1978) Thyroid disease and function in breast cancer patients and non-hospitalised controls evaluated by determination of TSH, $T_3$, r $T_3$ and $T_4$ levels in serum. *Acta Chir. Scand.*, **144**, 89.

ALDINGER, K. A., SCHULTZ, P. N., BLUMENSCHEIN, G. R. & SAMAAN, N. A. (1978) Thyroid stimulating hormone and prolactin levels in breast cancer. *Arch. Int. Med.*, **138**, 1638.

BRINCKMEYER, L. M., WORM, A. M. & NISSEN, N. I. (1977) Thyroid function in malignant lymphoma. *Acta Med. Scand.*, **202**, 475.

CHAN, V., WANG, C. & YEUNG, R. T. T. (1978) Pituitary-thyroid responses to surgical stress. *Acta Endocrinol.*, **88**, 490.

GORMAN, C. A., BECKER, D. V., GREENSPAN, F. S. & 5 others (1977) American Thyroid Association Statement: Breast cancer and thyroid hormone therapy. *Ann. Int. Med.*, **86**, 502.

MITTRA, I. & HAYWARD, J. L. (1974) Hypothalamic–pituitary thyroid axis in breast cancer. *Lancet*, **i**, 885.

ROSE, D. P. & DAVIS, T. E. (1978) Plasma thyroid stimulating hormone and thyroxine concentrations in breast cancer. *Cancer*, **41**, 666.

SCHIMMEL, M. & UTIGER, R. D. (1977) Thyroidal and peripheral production of thyroid hormones. *Ann. Int. Med.*, **87**, 760.

SHALET, S. M., BEARDWELL, C. G., MORRIS-JONES, P. H. & PEARSON, D. (1975) Pituitary function after treatment of intracranial tumours in children. *Lancet*, **ii**, 104.